# Human activities disturb haul out and nursing behavior of Pacific harbor seals at Punta Banda Estuary, Mexico

**DOI:** 10.1371/journal.pone.0270129

**Published:** 2022-07-06

**Authors:** María Guadalupe Ruiz-Mar, Gisela Heckel, Elena Solana-Arellano, Yolanda Schramm, María C. García-Aguilar, Maria Clara Arteaga

**Affiliations:** 1 Conservation Biology Department, Centro de Investigación Científica y de Educación Superior de Ensenada, Ensenada, Baja California, Mexico; 2 Marine Ecology Department, Centro de Investigación Científica y de Educación Superior de Ensenada, Ensenada, Baja California, Mexico; 3 Faculty of Marine Sciences, Universidad Autónoma de Baja California, Ensenada, Baja California, Mexico; 4 Biological Oceanography Department, Centro de Investigación Científica y de Educación Superior de Ensenada, Ensenada, Baja California, Mexico; Universidad de Antioquia, COLOMBIA

## Abstract

Humans frequently interact with Pacific harbor seals (*Phoca vitulina richardii*) at Punta Banda Estuary, Baja California, Mexico, due to the high incidence of recreational activities people undertake there. The immediate effect of these interactions is that seals flush to the water, reducing their time on land and, probably, increasing their energy expenditure. On-land observations were used to study the impact of different sources of disturbance on seal behavior and evaluate their effect on the amount of time dedicated to nursing over three pupping seasons, (2015–2017), with 0.58–0.81 disturbance events/hour recorded over the entire sampling period. Terrestrial vehicles were the source with the highest disturbance rate (number of disturbance events/h), followed closely by pedestrians. However, the proportion of seals affected was highest when pedestrians were the disturbance source. Recovery events (seals hauling out after flushing) occurred after 34% of disturbance events, after less than half of which the same number of hauled-out seals as there were prior to the disturbance were observed. Recovery time varied among the years studied, of which 2017 saw the longest recovery time. In addition, pedestrians were the disturbance source with the longest recovery time. Given that resting on land is essential for pup survival, which depends on both the establishment of the mother-pup bond from birth and its maintenance throughout nursing, flushing behavior may have significant implications for the entire colony during the nursing season. We recorded a decrease in nursing duration, which did not return to the same level even after recovery and the resumption of nursing. Terrestrial vehicles were found to be the disturbance source that shortened nursing events most significantly.

## Introduction

Pinnipeds are marine mammals which spend part of their annual cycle on land, in order to breed, molt, and rest [1]. As some haul-out sites are located very close to or even within human settlements, pinniped colonies may be threatened by human activities. Seals (family Phocidae) occur in different terrestrial habitats, such as sand and cobblestone beaches, rocky outcrops, sandbanks, and pack ice [1–3]. During the pupping season, haul-out sites are used by mother-pup pairs to nurse and rest [1]. During lactation and commencing 11 days after birth, Pacific harbor seal (*Phoca vitulina richardii*) females carry out up to seven foraging trips per day [4–6]. Rest is a physiological requirement during the molting season, in which seals replace fur, thus enabling nutrients, energy, and oxygen to be supplied to the epidermic cells while maintaining an optimal skin surface temperature [7] and minimal energy expenditure [8].

Among marine mammals, harbor seals are particularly susceptible to the effects of sea vessel and kayak traffic because of their wary, vigilant behavior and their reliance on nearshore haulouts often located in areas used by humans [9–13].

When disturbed, seals increase vigilance by raising their heads and then flushing to the water, reducing their time on land. This increases energy expenditure, or causes stress [7, 10]. An active response to disturbance causes physiological changes, such as increased heart and respiratory rate, body temperature, and blood glucose as well as reduced blood flow to the skin and digestive organs [14]. These occur in response to the release of two kinds of hormones: catecholamines (epinephrine and norepinephrine); and, glucocorticoids (cortisol in marine mammals) [15]. Cortisol contributes to the inhibition of certain types of behavior and reproductive physiology, facilitates movement responses, enhances memory related to both stress and learning, and increases the availability of energy [16]. However, it also has long-term (namely hours or days after the disturbance) effects in gene transcription and protein synthesis [17]. High levels of stress hormones may cause infertility, immunosuppression, weight and muscle loss, and growth inhibition, harm the central nervous system and cause deterioration in cognitive function [16]. Ultimately, disturbances may lead to reduced population numbers due to their impact on survival and reproductive success [18–20].

Mother-pup pairs are more sensitive to disturbances than other seals in the colony [9, 21–23]. As mother-pup pairs tend to rest either on the periphery of the colony or at separate sites, they are more vigilant, namely they need to spend more time scanning the area for possible threats than other seals situated in the center of the colony [24]. The larger the colony, the less time seals spend scanning [25]. Flushing behavior may affect the mother-pup relationship and may also cause pups to obtain less energy because they are forced to spend less time nursing, thus affecting their chances of survival [26–29].

In Mexico, the Pacific harbor seal occurs along the coast and on the islands to the west of the Baja California Peninsula, from the Coronado Islands to Asuncion Island [30]. Punta Banda Estuary (PBE), located on the edge of the urban area of the city of Ensenada, Baja California, is a wetland recognized by the Ramsar Convention and is also an important bird conservation area [31]. A small harbor seal colony of around 90 individuals hauls out at PBE near the mouth of the estuary, mainly during the pupping (February to April) and molting (end of April to mid-July) seasons [32]. Harbor seals are protected by law in Mexico via their inclusion on the Mexican list of at-risk species, in the lowest category of *Under Special Protection* [33]. In addition, the Federal Penal Code (*Código Penal Federal*) specifies, in its Paragraph 420, that anyone who catches, harms, or kills a marine mammal may be subject to a one-to-nine-year prison sentence and a fine [34].

Due to its proximity to the city of Ensenada, PBE is a site used for tourism and recreational activities. Although the disturbances caused by these activities to the seals resident at PBE have been previously recorded [32, 35, 36], their effects, at a colony level, are unknown. We hypothesized that the disturbance rate (number of disturbance events/h/day with a concomitant flushing reaction) at PBE would be higher than reported by previous studies on human disturbance at haul-out sites on the U.S. coast. In addition, we sought to test that recovery (defined in this context as the proportion of the group of seals that haul out after the disturbance) would occur after less than 50% of disturbance events observed. Furthermore, our hypothesis states that nursing duration may be reduced as a result of these disturbance events. Therefore, the present research aimed to study both the different disturbance sources and the seals’ behavioral responses, including the effect of disturbance on nursing duration.

## Methods

### Fieldwork

**Fig 1:**
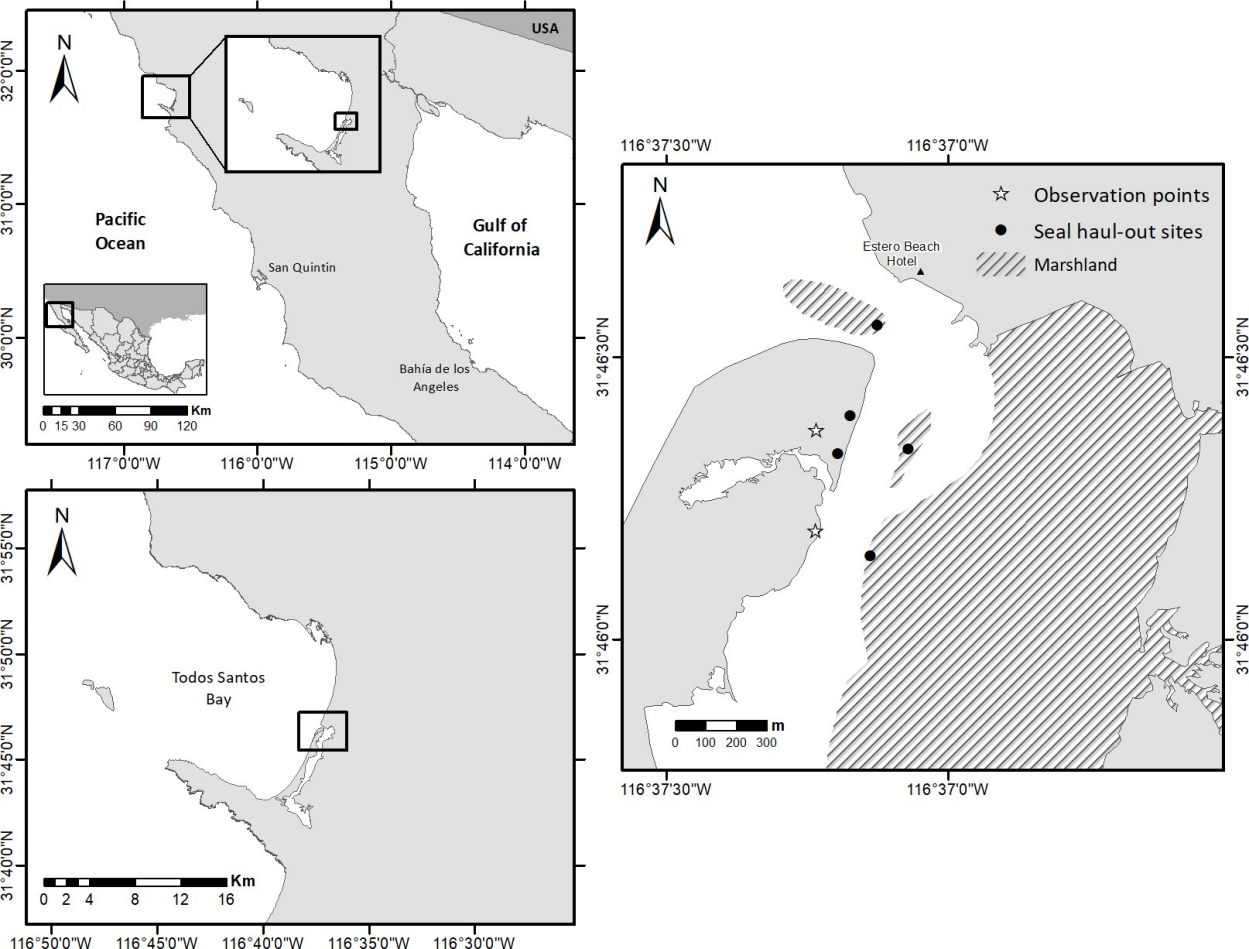
Location of study site: Punta Banda Estuary (PBE), Baja California, Mexico. Basemap source: CONABIO (*Comisión Nacional para el Conocimiento y Uso de la Biodiversidad*, National Commission on Biodiversity Knowledge and Use), *División política estatal 1*:*250*,*000* (State political division 1:250,000), http://www.conabio.gob.mx/informacion/metadata/gis/dest_2015gw.xml?_httpcache=yes&_xsl=/db/metadata/xsl/fgdc_html.xsl&_indent=no.

The present research was conducted at PBE, 13 km south of Ensenada, Baja California, Mexico (31°51’N, 116°38’W; Fig 1), during the 2015 (observation effort = 3 h/d), 2016 (observation effort = 4 h/d), and 2017 (observation effort = 4 h/d) pupping seasons, which occurred from February to April. As the study location has a semidiurnal tidal cycle, we chose to conduct our research at the lowest tide during daylight hours, as this is when the highest number of seals is hauled out [35, 37, 38]. Observations were carried out for the three to four hours during which low tide occurs and were conducted four days per week, while two sampling days were chosen at random each week. The Saturday and Sunday of each week were also sampled, given the higher potential for disturbance factors on weekends, which was also true during Easter week, a period of intense tourism activities in the estuary that was sampled in each of the three years in which this study was carried out. Using 7x50 binoculars and a telescope (20-60x80 mm), two researchers observed seals at two sites on the estuary’s sand bar (Fig 1), moving between observation sites according to the movements of the seals between the haul-out sites. As the observation sites were located approximately 200 m from the seals, we assumed that the observers did not effect the seals’ behavior. The behavioral observations were conducted *ad libitum* [39], namely that the haulouts were observed continuously with the seals’ behavioral responses recorded every time they occurred, while seal counts were undertaken every 15 minutes. A disturbance event was recorded when one or more animals flushed in response to a visual or auditory disturbance associated with some form of human activity. All haul-out sites were equally accessible to all the disturbance sources, which were recorded even if they did not elicit a behavioral response from the seals. However, if multiple disturbance sources were present, the disturbance that elicited a reaction was the one recorded.

Each disturbance event was recorded, along with the time of day and the following relevant details: the disturbance source; the number of seals that reacted to the disturbance; and, the recovery level (defined here as the proportion of seals that hauled out after the disturbance, assuming that they were from the same group that had flushed to the water). To assess the latter variable, seal counts were conducted every 15 min at all five haul-out sites. The maximum amount of time spent when determining whether recovery occurred corresponded to the complete low tide cycle that occurred on that sampling day, namely up to four hours.

The disturbance sources were classified as follows: motorized vessels (MV), such as small (< 5 m) boats and jet skis; vessels without motors (VWM), such as kayaks, paddle boards, and kiteboards; terrestrial vehicles (TV), such as motorcycles and trucks; noise (N), such as that generated by small airplanes, military airplanes, car alarms, and trucks; and, pedestrians (P), such as anglers carrying their equipment, clam collectors, or walkers. We were not able to measure the exact distance of the different sources to the seals, although we estimated that all, including TV, occurred within at least 100 m from the seals, at which distance the seals tended to flush.

At the start of each breeding season, we tagged pups by placing colored squares on their heads in order to carry out focal observations of the mother-pup pairs. They were selected according to their location within the colony (on the periphery, for example) and the position between mother and pup, to ensure that nursing could be observed with the telescope. Only one pup was observed at a time by the observer using the telescope.

In order to determine the effect of the disturbance sources on nursing, we recorded the duration, in minutes, of each nursing event, which was considered to have started when the pup placed its muzzle on its mother’s nipple and to have ended when the pup fell asleep and ceased to nurse without any apparent provocation or when it was disturbed. When the pup was disturbed, we recorded the source, whether it recovered, and whether it recommenced nursing.

Due to the lack of data obtained in 2017, we used the data from solely 2015 and 2016 for the analysis of the effect of disturbance on nursing duration, defining the three following scenarios:

Scenario 1 (S1)—although nursing is momentarily interrupted by a disturbance, the mother-pup pair does not flush and continues to nurse;
Scenario 2 (S2)—nursing is interrupted by a disturbance and the pair flushes, but recovers in less than 10 min and continues to nurse; and,
Scenario 3 (S3)—nursing is interrupted by a disturbance, the pair flushes, and nursing does not resume until the end of the low tide cycle occurring during the sampling effort conducted on that day.

The control observations comprised nursing events that were not interrupted because no disturbance source was present.

### Data analysis

Bayesian inference was used to analyze the data obtained by the present research. The analysis was conducted in JAGS (Just Another Gibbs Sampler) in R [40], which implements Bayesian inference by sampling from the posterior distribution of the parameter of interest via a Markov Chain Monte Carlo simulation. We ran three chains of 1,000,000 iterations, with a 10% warm-up phase and the tenth value retained. In most cases, non-informative (*i*.*e*. uniform) prior distributions were used.

#### The effects of disturbance on haul-out behavior

To analyze the tendency of disturbance over time, we estimated a disturbance rate (number of disturbances per hour for each sampling day). We compared the mean disturbance rates among the three years sampled with a Bayesian ANOVA, using the Bayes factor (BF), which computes the probability of the data observed under the null hypothesis against the alternative hypothesis [41].

In addition, we analyzed the change in the disturbance rate within each sampled year using simple Bayesian linear regression with a uniform prior.

In order to assess the seals’ reaction when disturbed, we calculated the proportion of seals that flushed after each event it order to compare it with the number of seals that remained hauled out. In order to avoid overestimating the proportion, we only took into account those flushing events that occurred when at least ten individuals (10% of the colony) were on land prior to the disturbance.

Given that the data was collected over a three-year period, we first evaluated the differences among sampling periods in terms of the proportion of the group of seals that reacted to the disturbance. We used Bayesian inference to obtain binomial proportions, assuming a prior *beta* (*a*,*b*) distribution, which is the conjugate family for binomial observation distribution [42]. As the posterior distribution of proportions must be higher than or equal to *beta* (10,10), the proportions are close to a normal distribution, meaning that paired comparisons can be carried out without the need for integration. We assumed *beta* prior distributions (1,1) for the three years, obtaining the following posterior distributions:

2015: *beta* (14,14)
2016: *beta* (11,16)
2017: *beta* (9,16)

As 2017 did not comply with said rule, we assumed a *beta* prior distribution (9,1), after which we carried out paired comparisons via a one-tailed hypothesis test. As differences among the years sampled were observed (see *Results*), the analyses were performed separately.

In order to determine the effect of disturbance sources on the number of seals engaging in flushing activity, we used the Bayesian inference again to calculate the binomial proportions, assuming non-informative prior distributions. We estimated the posterior distribution for each disturbance source and then conducted paired comparisons between sources, by means of one-tailed hypothesis tests.

Recovery was defined when at least one individual hauled out after the disturbance, while the variation in recovery time among the years sampled was evaluated via a Bayesian ANOVA test, assuming a uniform prior and a normal likelihood distribution. The same analysis was carried out to compare the variation in recovery time, by year, among the disturbance sources.

#### Effect of disturbance on nursing

We compared the mean duration of nursing events corresponding to the above-described scenarios with the control observations (see *Fieldwork* section), via a Bayesian ANOVA.

We also analyzed the disturbance effect using multidimensional scaling (MDS), in which the response variable was frequency, *i*.*e*. the number of times each scenario occurred. Multidimensional scaling is a spatial representation that refers to the geometric configuration of specific points, with the distance between points indicating the similarity between variables, wherein the closer the points, the greater the similarity among the variables they represent [43, 44]. We constructed two similarity matrices–the first for the three scenarios set out above and the second for each of the disturbance sources considered (MV, VWM, TV, N, and P). Both matrices conveyed a fused graph.

### Ethics statement

This study was carried out under permit No. SGPA/DGVS/1140/16, granted by the *Secretaría de Medio Ambiente y Recursos Naturales* (SEMARNAT, Environment and Natural Resources Ministry, SEMARNAT), Mexico.

## Results

### Effects of disturbance on haul-out behavior

In 2015, we conducted 41 effort days, which comprised 110 hours of observing seal behavior, recording 50 disturbance events in response to which the seals presented a high intensity reaction, *i*.*e*. flushing, at a mean rate of 0.81 events/h/day (95% Credibility Interval CI = 0.53–1.1). In 2016, we conducted 32 effort days, which comprised 127 observation hours, recording 68 disturbance events with a flushing response at a mean rate of 0.58 events/h/d (95% CI: 1–1.77). In 2017, we conducted 38 effort days, which comprised 136 observation hours, recording 79 disturbance events with a flushing response at a mean rate of 0.71 events/h/day (95% CI = 0.48–0.96). According to a Bayesian ANOVA, there were significant differences between 2015 and 2016 (BF = 191) and 2016–2017 (BF = 1249), but not between 2015 and 2017 (BF = 2.2)

**Fig 2:**
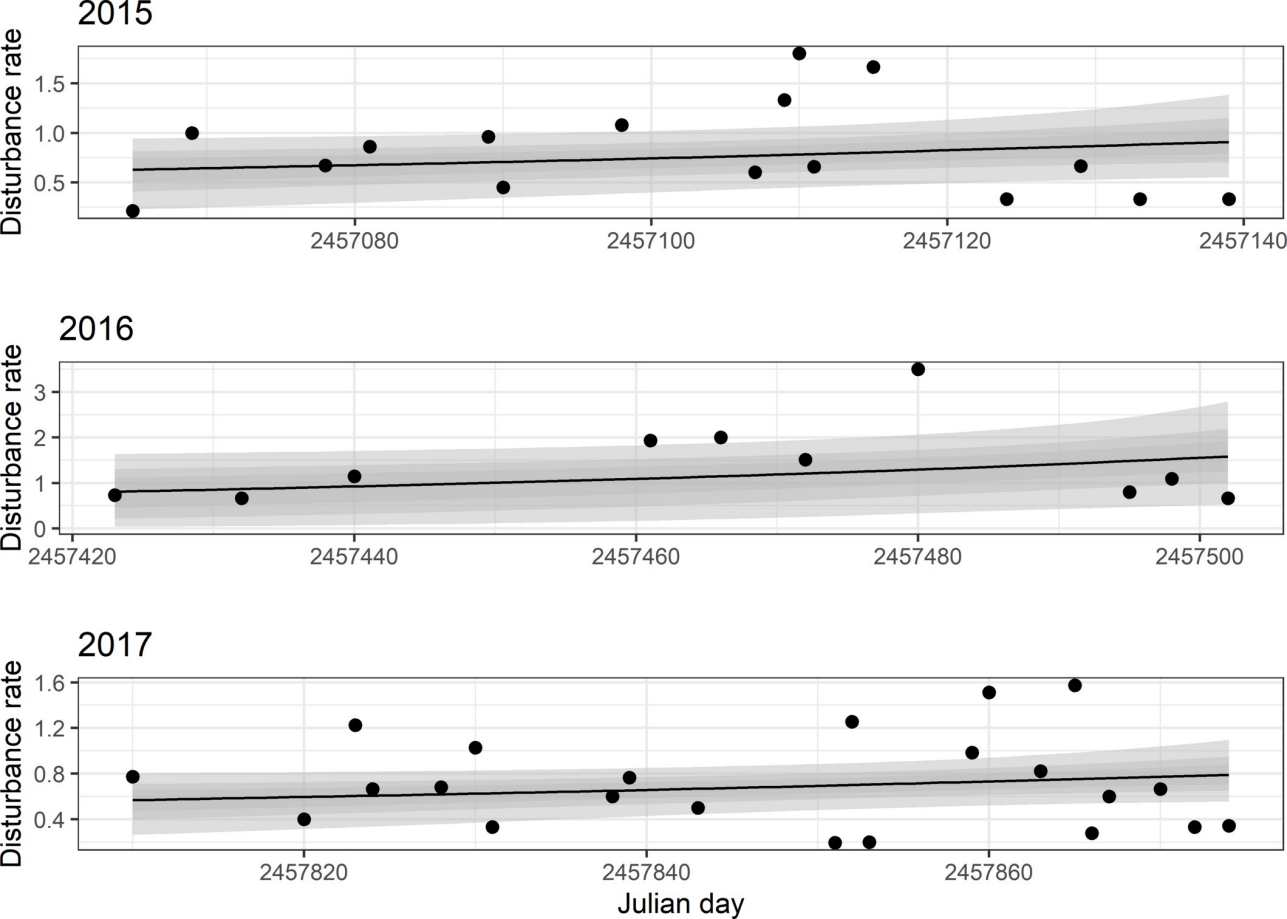
Bayesian linear regressions of disturbance rates (number of disturbance events/h) for the sampling periods undertaken in 2015, 2016, and 2017. Shaded areas correspond to 95% Bayesian credible intervals.

For all three years, the disturbance rate increased throughout each sampling period (Fig 2). In 2015, the regresion slope was 0.1 (95% CI:0.04–0.45); in 2016, 0.22 (95% CI = -0.01–1.29; and, in 2017, 0.09 (95% CI = 0.004–0.37).

As we found credible differences, among the years sampled (BF>33 –Supporting information: S1 and S2 Tables), in terms of the posterior proportions of the high intensity reactions observed, the subsequent analyses were performed independently for each year sampled.

**Fig 3:**
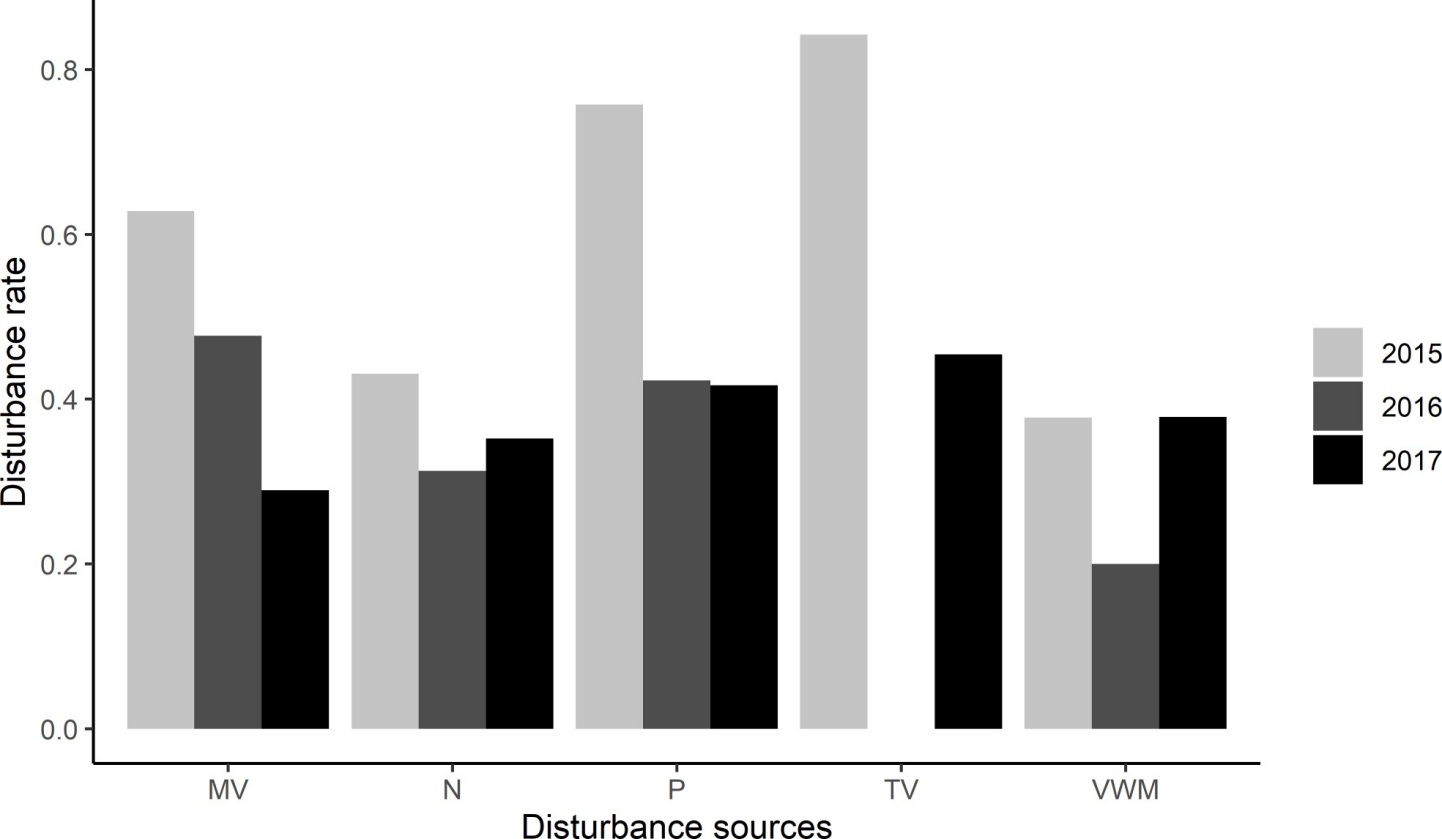
Disturbance rates (number of disturbance events/h) at PBE over three breeding seasons (white—2015, grey—2016, and black—2017). Disturbance sources: P = pedestrians; MV = motorized vessels; VWM = vessels without motor; TV = terrestrial vehicles; and, N = noise.

Terrestrial vehicles (TV) were the source with the highest disturbance rate (number of disturbance events/h), followed closely by pedestrians (P) in 2015 and 2017. Motorized vessels (MV) had the highest disturbance rate in 2016 (Fig 3).

The proportion of seals affected was highest when P was the disturbance source (Table 1), presenting credible differences when compared with all other sources (MV, VWM, TV, and N), except between P and MV in 2016 (Table 2). In 2017, VWM affected a higher proportion of seals than MV and N (Tables 1 and 2).

**Table 1:** Posterior summaries for the proportion of seals presenting a high intensity reaction, *i*.*e*. flushing, to a disturbance source.

Disturbance					Credible interval	
source	Year	n	Mean	SD	2.5%	97.5%
P	2015	17	0.58	0.03	0.53	0.63
	2016	27	0.46	0.02	0.43	0.50
	2017	25	0.58	0.02	0.54	0.62
MV	2015	6	0.23	0.11	0.06	0.49
	2016	14	0.43	0.03	0.38	0.49
	2017	11	0.24	0.03	0.19	0.30
VWM	2015	7	0.29	0.03	0.23	0.35
	2017	7	0.36	0.04	0.29	0.43
TV	2015	4	0.26	0.06	0.16	0.37
	2017	8	0.27	0.04	0.20	0.35
N	2016	6	0.15	0.03	0.10	0.21
	2017	13	0.19	0.02	0.15	0.23

Disturbance sources: P = pedestrians; MV = motorized vessels; VWM = vessels without motor; TV = terrestrial vehicles; and, N = noise. SD = standard deviation, n = sample size Not all disturbance sources were compared due to a lack of data.

**Table 2:** Bayes factor comparison between pairs of disturbance sources based on the posterior proportion of disturbed seals presenting a high intensity reaction, *i*.*e*. flushing. Numbers in bold indicate credible differences. The probability that the first disturbance source was higher than the second one is also shown.

Disturbance sources	Year	Bayes factor	Probability	Disturbance sources	Year	Bayes factor	Probability
P_TV	2015	**>100**	**1**	MV_N	2016	**>100**	**1**
	2017	**>100**	**1**		2017	**15.5**	**0.93**
P_MV	2015	**>100**	**1**	VWM_MV	2015	**5.3**	**0.84**
	2016	**4.45**	**0.88**		2017	**>100**	**0.99**
	2017	**>100**	**1**	VWM_N	2017	**>100**	**1**
P_VWM	2015	**>100**	**1**	VWM_TV	2015	**10.11**	**0.68**
	2017	**>100**	**1**		2017	**24.79**	**0.96**
P_N	2016	**>100**	**1**	MV_TV	2015	0.008	0.40
	2017	**>100**	**1**		2017	0.4	0.29
				TV_N	2017	**36.32**	**0.97**

Disturbance sources: P = pedestrians; MV = motorized vessels; VWM = vessels without motor; TV = terrestrial vehicles; and, N = noise. Not all disturbance sources were compared due to lack of data.

The seals studied recovered after 34% of disturbance events, with a return to pre-disturbance levels observed in 42% of said events. The mean recovery time recorded in 2017 (16.38 min, 95% CI = 1.08–38.7) was shorter than that recorded in either 2015 (32.34 min, 95% CI = 11–54.19) or 2016 (35.92 min, 95% CI = 17.24–54.49), for which BFs of 11 and 6 were calculated, respectively. However, no credible differences were observed between the mean recovery times recorded in 2015 and 2016 (BF = 0.66).

Due to the scarcity of data, we analyzed the recovery time after three specific disturbance sources were recorded: MV; VWM; and, P (Table 3). In 2016 and 2017, the seals’ recovery time in response to P disturbance sources was longer than that observed in response to disturbances caused by MV (BF = 14 and 4, respectively) (Tables 3 and 4).

**Table 3:** Posterior summaries for recovery duration (min), by disturbance source.

					Credible interval	
Disturbance sources	Year	n	Mean	SD	2.5%	97.5%
P	2015	6	39.08	29.5	-20.41	98.72
	2016	8	59.67	16.55	25.33	91.04
	2017	6	16.72	7.72	1.42	32.54
MV	2016	7	22.3	18.54	-14.68	59.22
	2017	3	5.93	11.02	-15.9	28
VWM	2015	3	32.15	48.08	-63.92	133.99

P = pedestrians, MV = motorized vessels, VWM = vessels without motor. SD = standard deviation, n = sample size. Not all disturbance sources were compared due to a lack of data.

**Table 4:** Bayes factor for differences between the posterior means calculated for the duration of recovery, by disturbance source. Numbers in bold indicate credible differences. The probability that the first disturbance source was higher than the second one is also shown.

Disturbance sources	Year	Bayes factor	Probability
P_MV	2016	**14.4**	**0.93**
	2017	**4.33**	**0.81**
P_VWM	2015	1.27	0.57

P = pedestrians, MV = motorized vessels, VWM = vessels without motor. Not all disturbance sources were compared due to a lack of data.

While we analyzed the possible effect of group size on the proportion of seals that either flushed or recovered, regression analyses showed a slope close to 0. Therefore, group size does not seem to have any effect on flushing or recovery behavior.

### Effect of disturbance on nursing duration

**Fig 4:**
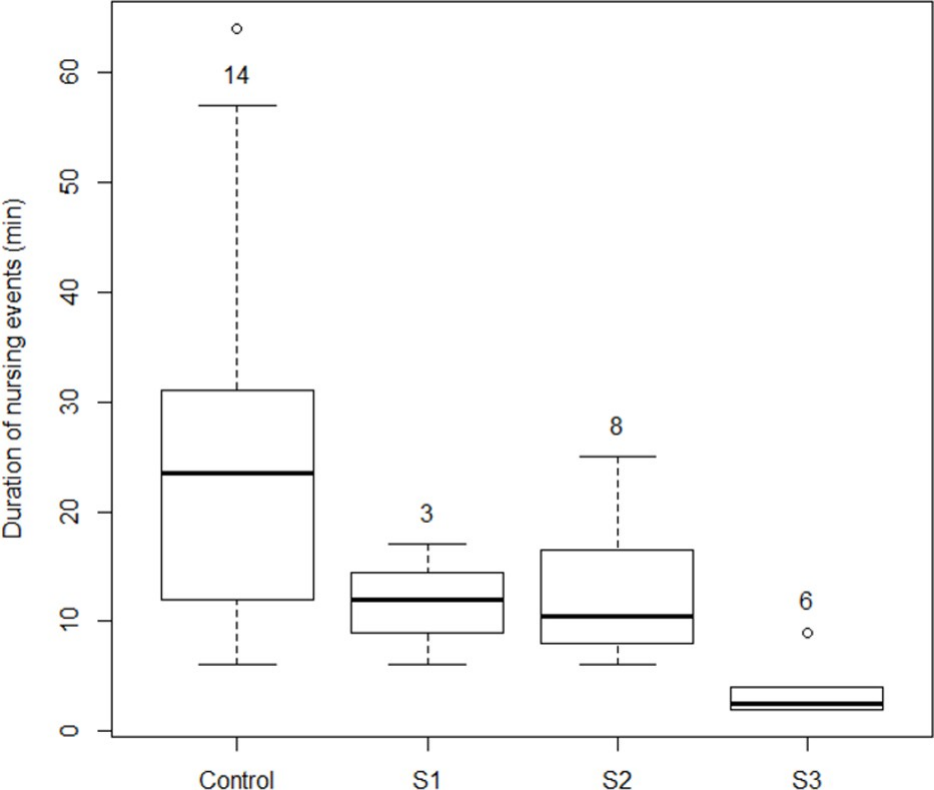
Nursing duration (min) under different disturbance scenarios and the control (no disturbance). Boxplots depict mean (horizontal line), standard deviation (box), and maximum and minimum values (bars). Circles denote outliers. Numbers correspond to the number of observations for each scenario. Scenario 1 (S1)–although nursing is momentarily interrupted by a disturbance, the mother-pup pair does not flush and continues to nurse; Scenario 2 (S2)–nursing is interrupted by a disturbance and the pair flushes, but recovers in less than 10 min and continues to nurse; and, Scenario 3 (S3)–nursing is interrupted by a disturbance, the pair flushes, and nursing does not resume until the end of the low tide cycle occurring during the sampling effort conducted on that day. The control observations (C) comprised nursing events that were not interrupted because no disturbance source was present.

We analyzed the effect of the disturbances on nursing duration under the three observation scenarios set out above (S1, S2, and S3) and a control (in which no disturbance occurred). The most frequent disturbance scenario observed was S2 (Fig 4), while the mean nursing duration in all three scenarios (S1–12.8 min, S2–12.7 min, and S3–3.8 min) was shorter than that recorded in the control scenario (26.7 min, BF>20) (Table 5 and Fig 4). However, no credible differences were observed in the mean nursing duration between S1 and S2 (BF = 0.96), which were longer than that recorded for S3 (BF = 6 and 8, respectively) (Table 4 and Fig 4).

**Table 5:** BF for differences in nursing duration between the disturbance scenarios and control observations (in which no disturbance occurred). The probability that the nursing duration in the first scenario was longer than in the second one is also shown. Scenario 1 (S1)–although nursing is momentarily interrupted by a disturbance, the mother-pup pair does not flush and continues to nurse; Scenario 2 (S2)–nursing is interrupted by a disturbance and the pair flushes, but recovers in less than 10 min and continues to nurse; and, Scenario 3 (S3)–nursing is interrupted by a disturbance, the pair flushes, and nursing does not resume until the end of the low tide cycle occurring during the sampling effort conducted on that day. The control observations (C) comprised nursing events that were not interrupted because no disturbance source was present.

Disturbance scenarios	Bayes factor	Probability
S1_S2	0.96	0.49
S1_S3	**5.54**	**0.85**
S2_S3	**8.31**	**0.89**
C_S1	**20.53**	**0.95**
C_S2	**82.51**	**0.99**
C_S3	**>100**	**0.99**

**Fig 5:**
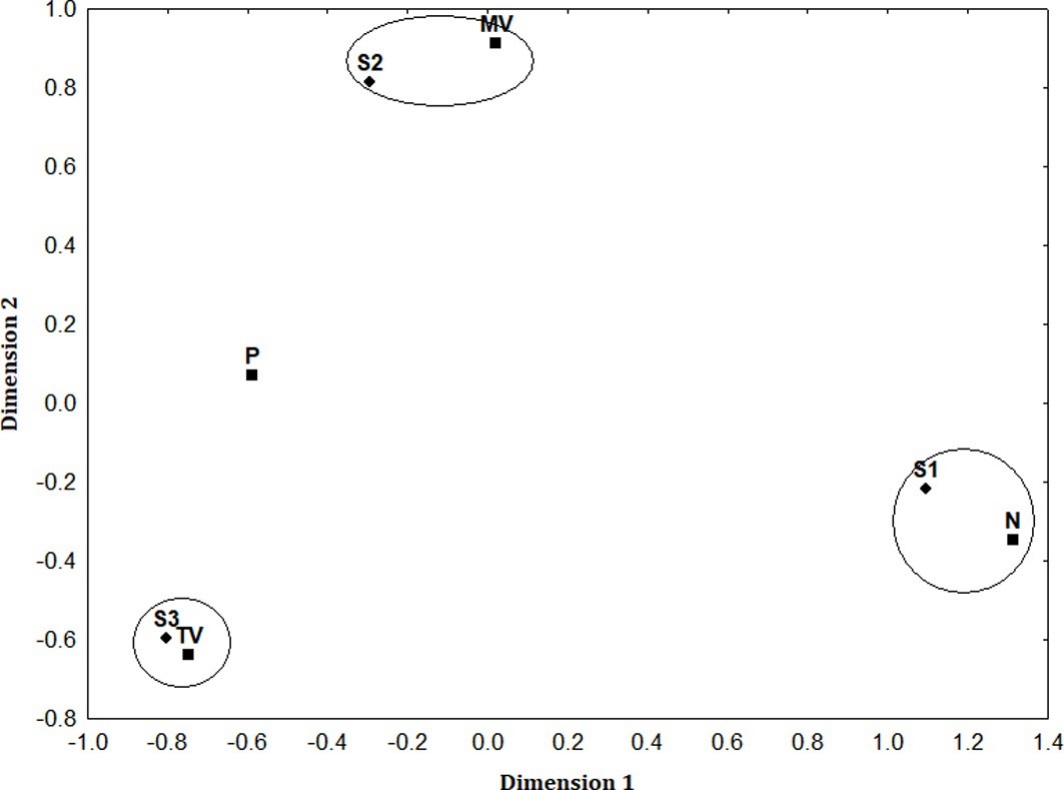
Relationships between disturbance sources and mother-pup reaction scenarios, according to multidimensional scaling. Scenario 1 (S1)–although nursing is momentarily interrupted by a disturbance, the mother-pup pair does not flush and continues to nurse; Scenario 2 (S2)–nursing is interrupted by a disturbance and the pair flushes, but recovers in less than 10 min and continues to nurse; and, Scenario 3 (S3)–nursing is interrupted by a disturbance, the pair flushes, and nursing does not resume until the end of the low tide cycle occurring during the sampling effort conducted on that day. The control observations (C) comprised nursing events that were not interrupted because no disturbance source was present.Disturbance sources: P = pedestrians; MV = motorized vehicles; N = noise; and, TV = terrestrial vehicles.

We also analyzed the relationships between disturbance sources and mother-pup reaction scenarios via MDS (Fig 5), finding that S1 was most similar to disturbance source N (*i*.*e*., they are in close proximity), while S2 is most similar to disturbance source MV and S3 is most similar to disturbance source TV. These MDS results mean that disturbance source N interrupted nursing events, but did not elicit flushing, with nursing continuing sometime after, while disturbance source MV did elicit flushing, after which the seals hauled out and then resumed nursing. TV were the disturbance source that most affected the mother-pup pairs, interrupting nursing events and eliciting a flushing reaction that was not followed by hauling out and a resumption of nursing.

## Discussion

### Effects of disturbance on haul-out behavior

Wildlife tends to react and flee from the presence of human beings, given that they have hunted animal species for hundreds or thousands of years [45]. In the three years that comprised the present study, we recorded 0.58–0.81 yearly mean disturbance events/h at the lowest tide during daylight hours, a higher disturbance rate than that recorded at haul-out sites in the San Francisco Bay area (California, USA): 0.17 at Corkscrew Slough; 0.38 at Yerba Buena Island; and, 0.44 at Castro Rocks [46]. In addition, the disturbance rate increased slightly during the entire pupping season in the years sampled, which shows that this estuary is intensively used for human activities. However, it is likely that not all visitors to the area cause a disturbance, which we did not measure and recommend should be explored in future studies.

As flushing events evidently interrupt seals’ resting and nursing behavior, investment in avoidance behavior will reduce energy intake, damage body condition, and limit reproductive success, according to the risk-disturbance hypothesis [47]. These effects are exacerbated when environmental conditions are unfavorable (e.g. low prey availability during El Niño conditions) and may lead, ultimately, to a reduction in population size [47]. The PBE harbor seal colony has a small population size (around 90 individuals), which was first counted during the 1980s [32, 35, 36]. This small size may be related to both the ecological carrying capacity of the estuary and the continuing human disturbance to which the seal colony has been exposed for decades.

Foraging at night, harbor seals spend more daylight than nocturnal hours on land, meaning that they are more exposed to human disturbance during the day [48]. Our study found that the individuals at PBE remain on land at low tide (when their haul-out sites are accessible) for the six-hour period that elapses until high tide [49].

Analysis of the disturbance sources found that TV and P elicited the highest proportion of flushed seals. As harbor seals move slowly on land via a hitching motion of the body [50], they may interpret a disturbance on land as a greater threat than a disturbance in the water. While the different P groups (anglers, clam collectors, and walkers) behaved in a slightly different way, the reaction of the seals was the same, namely flushing. Anglers and clam collectors seem to perceive that the best sites for collecting clams or fishing were where seals haul out, approaching the colony directly in order to cause the seals to flush and then beginning to fish or dig for clams. Walkers also approached the seals directly, most likely out of simple curiosity and ignorance about their instinctive reaction. In terms of the proportion of flushed seals, P was the most significant disturbance source, although no differences were found between it and MV in 2016 (when the proportion of flushed seals in response to MV was as high as that observed in response to P), probably due to a high presence of boats and jet skis in the estuary that year. P have also been reported as a significant disturbance source for harbor seal colonies in the Dollard Estuary, the Netherlands, and Glacier Bay, Alaska [29, 51].

Harbor seals present flushing behavior when pedestrians are within a distance of 100 m [9, 51, 52]. Although our study did not analyze the distance at which the harbor seals reacted to disturbance sources, we were able to observe that the animals flushed at approximately that distance. In contrast, elephant seals (*Mirounga leonina* and *M*. *angustirostris*) present unusually robust responses to human disturbance, with visitors to the Año Nuevo Nature Reserve, California, permitted to approach northern elephant seals up to a minimum of 8 m, even at which distance the presence of humans does not disturb them [53].

At PBE, we found that recovery corresponded to 34% of seals that had hauled out after a disturbance. A factor that may influence recovery may be the length of time that a disturbance source remains in the area. Even though we did not measure this, our field experience reveals that the seals did not haul out for as long as the disturbance source, *e*.*g*. a clam collector, remained at the site. Instead, the seals would haul out at a different site or leave the estuary entirely. In addition, a return to pre-disturbance levels was observed after only 42% of disturbance events. Seal recovery has been recorded at 66% at Bolinas Lagoon, California [52] and at 50% on Yellow Island, Washington [11].

At our study site, recovery time varied among the years sampled, with an average 3 min observed in 2015 and 2016 and 16 min observed in 2017, which coincides with the varying average recovery time reported for seal colonies in the literature: 28 min at Bolinas Lagoon; 35 min on Yellow Island; and, 46 min at both Clements Reef and on Puffin and Skipjack islands [11, 52, 54]. In southwest Britain, grey seals (*Halichoerus grypus*) were not found to recover (i.e. they did not haul out again during a tide period) when private motor boats approached their haul-out sites too quickly (>5 kn) and at too close a proximity (<25 m) [55].

We observed that the individuals studied at PBE took longer to recover when P, rather than MV, was the disturbance source. In some of the years sampled, VWM caused a higher proportion of seals to flush than MV. As kayaks are able to approach seals at a closer proximity than motorboats and do not produce distinctive sounds that would indicate their approach, they may be perceived by seals as predators that approach slowly and silently underwater [10]. Other studies analyzing the impact of vessels on harbor seal colonies have reported that MV elicit a lower number of flushing events than VWM at the following locations in the United States and Canada: Bolinas Lagoon [52] and Bair Island [46], California (USA); Puffin and Skipjack islands and Clements Reef [9] and Yellow Island [11], Washington (USA); and, McBride Glacier Fjord, Canada [51].

On an individual level, recovery time may depend on each seal’s energy budget and its current stage in the life cycle. Harbor seals have been observed to be more tolerant to disturbance during molting than during breeding [10], while Suryan and Harvey (1999) determined that the main factors affecting recovery were resting time and the number of hauled-out seals. Another factor that may influence recovery time is the availability of haul-out sites, where limited sites post-disturbance may result in a change in the nocturnal use of the initial site or its abandonment (either temporary or permanent) [10, 52, 56].

Seals may, however, stay in disturbed areas because they have become habituated to disturbance [46, 56] and choose haul-out sites close to locations where their potential prey occurs [57, 58].

### Effect of disturbance on nursing duration

Undisturbed harbor seal mothers typically remain in close contact with their pups, with separations rarely occurring until a pup is weaned [6, 59]. In our study, nursing events at PBE were disturbed more often than not. The response of mother-pup pairs to disturbance was characterized by shorter nursing times, even when nursing resumed during low tide, than those observed when no disturbance occurred, a finding assumed, but not explored, in previous studies [26, 29]. In addition, the shortest nursing time we recorded at PBE occurred under S3, in which mother-pup pairs flush in response to a disturbance event and do not resume nursing. In the Dollard Estuary, harbor seals have been found to undergo disturbance for one hour during every low tide period, which corresponds to approximately 12.5% of the time required for suckling [60]. Harp seals (*Phoca groenlandica*) in the Gulf of St. Lawrence, Canada, have also been observed to reduce nursing time in the presence of tourists on land [61]. In a controlled experiment, lactating Weddell seals (*Leptonychotes weddellii*) were observed to become habituated, namely presenting a less intense response, after ten pedestrian approaches within two hours. However, over a period of three weeks characterized by irregular pedestrian approaches, both mothers and pups presented sensitization instead of habituation [62].

The disturbance sources at PBE elicited different reaction scenarios, wherein N seems to be perceived by seals as a low-level threat, given that nursing was interrupted, but the mother-pup pairs did not flush and continued nursing (S1). Similar to the reactions presented by the whole colony to TV, this disturbance source elicited the worst reaction in the mother-pup pairs, wherein they flushed and did not resume nursing (S3). MV also elicited flushing, although less than 10 min later, the mother-pup pairs had hauled out again and resumed nursing (S2). As explained above in the context of the flushing reactions of the whole colony (adults and subadults), mother-pup pairs are also more vulnerable on land and, therefore, present a more extreme reaction to TV.

The timing of the disturbance relative to the tidal state could be expected to have an impact on nursing duration. If a disturbance occurs early in the low tide period, mother-pup pairs may haul out again within the same tidal cycle. However, we did not analyze this and recommend that this be explored in future studies.

Due to the milk energy output, which is a function of both the composition and the amount of milk produced, the duration of each suckling session and the suckling frequency can be useful indices for estimating energy transfer in phocids [63]. The composition of the milk produced by harbor seals changes significantly over the course of the lactation period, with the fat content increasing significantly from Day 0 to Day 7 and then remaining relatively constant throughout the remainder of the lactation period [64]. The composition of the milk produced by females that have lost or become separated from their pups for more than two days changes dramatically, with a 20%-23% decline in milk fat and a 6%-11% increase in protein [64]. If the effect of separation on milk composition in harbor seals is as rapid as that reported by Lang and milk output is affected by milk stasis in the same way as it is in other species, the separation of mother-pup pairs may be reflected in the level of energy transfer to the pup in terms of not only a reduction of suckling frequency but also the consequent reductions in both milk energy content and milk output [64].

The foregoing is, in turn, a significant determinant of pup mass gain [65, 66]. Therefore, when the time invested in nursing is reduced, the energy transferred from mother to pup will decrease. Consequently, the usual 0.3–0.7 kg/d increase in pup mass will not occur [67, 68], while pup mass tends to decline in pups soon to begin weaning [69]. Disturbances can effect harbor seal pups during every stage of lactation, given that the duration of suckling sessions has been observed to increase in line with pup age in harbor seals [6, 49, 70], northern and southern elephant seals [71, 72], harp seals [73], and grey seals [74]. This finding has energetic implications for the different stages of harbor seal lactation [74], wherein a reduction in the time spent suckling has been observed to increase mortality [69], in that the pup’s weight at weaning may be insufficient for its survival, with the mother opting not to invest more energy in order to avoid compromising her future reproductive capability [69, 75].

Another influence on pup survival is disturbance-induced stress, which may increase susceptibility to disease [76]. Furthermore, flushing due to disturbance may separate mother and pup permanently if their bond of mutual recognition is yet to be established, resulting in the death of the newborn due to emaciation and the thermal stress resulting from being forced to stay in the water for an unnecessarily long time [6, 29, 35, 77]. At PBE, we observed nine permanent mother-pup separations: two in 2015; one in 2016; and, six in 2017.

### Conclusions and management recommendations

From February to April (the pupping season), the high incidence of recreational activities at PBE disturbs its seal colony, whose members flush to the waterand at an even greater intensity when terrestrial vehicles and pedestrians approach. Moreover, disturbance decreases nursing time and, therefore, energy input, which may compromise pup survival.

Disturbances to harbor seals and the associated increases in energetic costs may have population-level effects by means of changes to pup recruitment that result from an earlier wean date and a lighter wean mass [48], decreased survival [77, 78], disruptions during breeding and pup rearing [9, 20, 22, 79], and relocation [10, 52, 80]. Among marine mammals, harbor seals are particularly susceptible to the effects of human activities because of their wary, vigilant behavior and their reliance on nearshore haul-outs often located in areas accessed by humans [9–13]. In order to maintain healthy populations, seals need quiet places where they can haul out and suckle their pups [60].

The management of human activities, commonly tourism, is often proposed as a measure to tailor disturbance reduction to specific pinniped species and their haul-out sites, as behavioral responses vary from species to species. Therefore, management measures have to be implemented on a case-by-case basis. As in many situations in which human activities and nature meet, effective management requires a combined educational and law enforcement approach.

**Fig 6:**
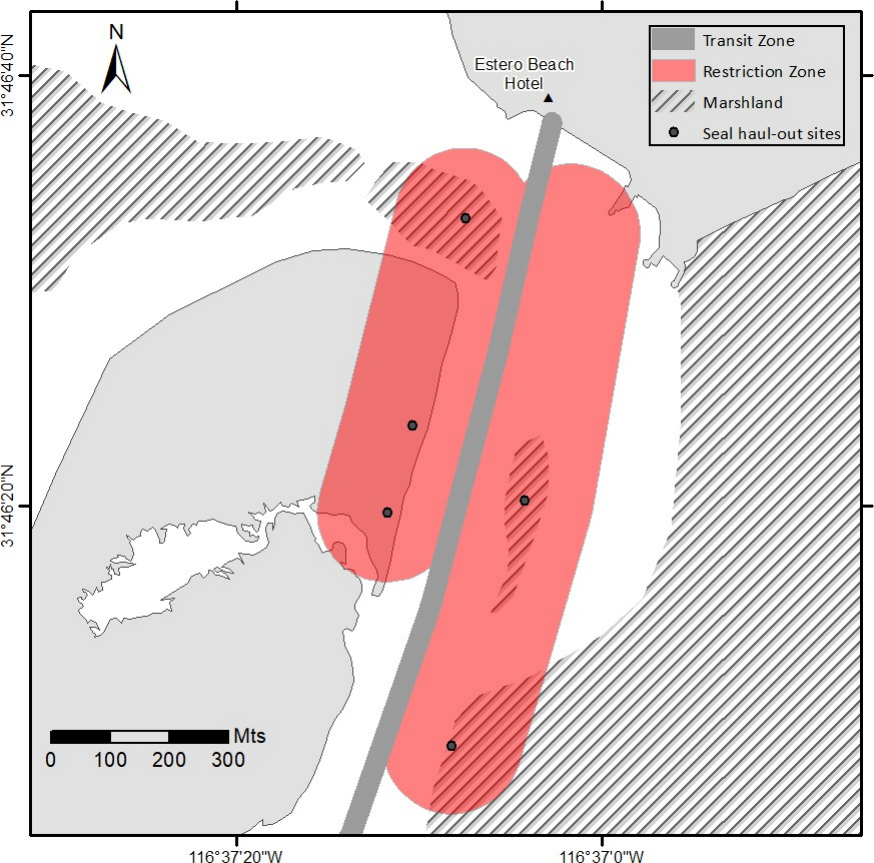
Zoning proposal for PBE with the objective of restricting both land and sea access to the harbor seal haul-out sites. The strategy proposed would be enforced by local authorities during the pupping season, from February to April, each year. Basemap source: IMIP (*Instituto Metropolitano de Investigación y Planeación de Ensenada*, *Baja California*, or Ensenada Metropolitan Research and Planning Institute, Baja California), “*SIG y mapas en línea*”–“*Imágenes digitales de alta resolución (línea de costa-El Sauzal)*”, or GIS and online maps–High resolution digital images (El Sauzal coastline). http://sigme.imipens.org/LC/.

Implementing the following recommendations at PBE would both mitigate the impacts on its seal colony and aid its conservation, recommendations similar to which have been proposed for harbor seals at Yellow Island, Washington [11]:

- Environmental education campaigns should be conducted for the residents of Punta Banda and guests at the Estero Beach Hotel (the area’s main disturbance source), involving talks on harbor seal biology, the importance of the harbor seal to the ecosystem, and the effects of disturbance during the pupping season.
- As harbor seals are protected by law (see *Introduction*), the Mexican environmental protection agency (PROFEPA, for its acronym in Spanish) should protect the nursing area from February to April every year by implementing a surveillance program.
- Humans should maintain, from both the water and on land, a safe distance of 100 m from hauled-out harbor seals.
- Advisory signs should be placed at strategic points to ensure that people both are aware of the potential impact of their activities on seals and respect the minimum safe distance stated above.
- As stakeholder-agreed management plans are more effective than command-control management strategies, local, regional, and national authorities should establish contact with both Punta Banda residents and the Estero Beach Hotel to regulate the tourism activities undertaken near haul-out sites.
- To facilitate the establishment of a management plan for the harbor seal population during the pupping season (February to April), we propose zones with restricted access to haul-out sites and a transit zone for vessels (Fig 6).

## Supporting information

S1 Table Posterior summaries for the proportion of high intensity reactions, by sampling year. SD = standard deviation.
(DOCX)

S2 Table Bayes factor for differences between the posterior proportions of the high intensity reactions, by sampling year. SD = standard deviation. The probability that the first year was higher than the second one is also shown.
(DOCX)
