# Supplementary material for: Human activities disturb haul out and nursing behavior of Pacific harbor seals at Punta Banda Estuary, Mexico
Source: PLoS One. 2022 Jul 6;17(7):e0270129. doi: 10.1371/journal.pone.0270129 (PMC9258837; doi:10.1371/journal.pone.0270129)
Supplement: S1 Table — SD = standard deviation. (DOCX) [file pone.0270129.s001.docx]

|  |  |  | **Credible interval** | |
| --- | --- | --- | --- | --- |
| **Year** | **Mean** | **sd** | **2.5%** | **97.5%** |
| 2015 | 0.40 | 0.02 | 0.37 | 0.43 |
| 2016 | 0.40 | 0.01 | 0.37 | 0.42 |
| 2017 | 0.36 | 0.01 | 0.34 | 0.39 |
