# Supplementary material for: Human activities disturb haul out and nursing behavior of Pacific harbor seals at Punta Banda Estuary, Mexico
Source: PLoS One. 2022 Jul 6;17(7):e0270129. doi: 10.1371/journal.pone.0270129 (PMC9258837; doi:10.1371/journal.pone.0270129)
Supplement: S2 Table — SD = standard deviation. The probability that the first year was higher than the second one is also shown. (DOCX) [file pone.0270129.s002.docx]

| **Year** | **Mean** | **sd** | **Bayes factor** | **Probability** |
| --- | --- | --- | --- | --- |
| 2015-2016 | 0.05 | 0.02 | **>100** | **0.79** |
| 2016-2017 | 0.040 | 0.02 | **33.05** | **0.84** |
| 2015-2017 | 0.04 | 0.02 | **42.85** | **0.96** |
